# TPL Inhibits the Invasion and Migration of Drug-Resistant Ovarian Cancer by Targeting the PI3K/AKT/NF-κB-Signaling Pathway to Inhibit the Polarization of M2 TAMs

**DOI:** 10.3389/fonc.2021.704001

**Published:** 2021-07-26

**Authors:** Fuyin Le, Lilan Yang, Yiwen Han, Yanying Zhong, Fuliang Zhan, Ying Feng, Hui Hu, Tingtao Chen, Buzhen Tan

**Affiliations:** ^1^ Department of Obstetrics & Gynecology, The Second Affiliated Hospital of Nanchang University, Nanchang, China; ^2^ Department of Obstetrics & Gynecology, The First Affiliated Hospital of Nanchang University, Nanchang, China; ^3^ Institute of Translational Medicine, Nanchang University, Nanchang, China

**Keywords:** A2780/DDP cells, cisplatin (DDP) resistance, triptolide (TPL), PI3K/AKT/NF-κB- pathway, tumor-associated macrophages (TAMs)

## Abstract

Chemoresistance is the primary reason for the poor prognosis of patients with ovarian cancer, and the search for a novel drug treatment or adjuvant chemotherapy drug is an urgent need. The tumor microenvironment plays key role in the incidence and development of tumors. As one of the most important components of the tumor microenvironment, M2 tumor-associated macrophages are closely related to tumor migration, invasion, immunosuppressive phenotype and drug resistance. Many studies have confirmed that triptolide (TPL), one of the principal components of *Tripterygium wilfordii*, possesses broad-spectrum anti-tumor activity. The aims of this study were to determine whether TPL could inhibit the migration and invasion of A2780/DDP cells *in vitro* and *in vivo* by inhibiting the polarization of M2 tumor-associated macrophages (TAMs); to explore the mechanism(s) underlying TPL effects; and to investigate the influence of TPL on murine intestinal symbiotic microbiota. *In vitro* results showed that M2 macrophage supernatant slightly promoted the proliferation, invasion, and migration of A2780/DDP cells, which was reversed by TPL in a dose-dependent manner. Animal experiments showed that TPL, particularly TPL + cisplatin (DDP), significantly reduced the tumor burden, prolonged the life span of mice by inhibiting M2 macrophage polarization, and downregulated the levels of CD31 and CD206 (CD31 is the vascular marker and CD206 is the macrophage marker), the mechanism of which may be related to the inhibition of the PI3K/Akt/NF-κB signaling pathway. High-throughput sequencing results of the intestinal microbiota in nude mice illustrated that *Akkermansia* and *Clostridium* were upregulated by DDP and TPL respective. We also found that *Lactobacillus* and *Akkermansia* were downregulated by DDP combined with TPL. Our results highlight the importance of M2 TAMs in Epithelial Ovarian Cancer (EOC) migration ability, invasiveness, and resistance to DDP. We also preliminarily explored the mechanism governing the reversal of the polarization of M2 macrophages by TPL.

## Introduction

Ovarian cancer is the leading cause of death among all gynecological malignances, and chemoresistant ovarian cancer is the principal cause of poor healing in patients (1, 2). Considering the shortcomings of current treatment modalities for ovarian cancer, including cytoreductive surgery and platinum-taxane combined chemotherapy, it is of paramount importance to develop novel strategies to treat this disease.

The occurrence and development of drug-resistant ovarian cancer is a complex and multifactorial process, involving the tumor microenvironment (3), matrix metalloproteinases (4), the epithelial-mesenchymal transition (5), and autophagy (6). Of these, the tumor microenvironment is closely related to tumor invasion and metastasis, and significantly affects the efficiency and effectiveness of tumor treatment (7). The tumor micro- environment is composed of tumor cells, the surrounding tissue fluid, cytokines, and stromal cells, including various immune cells, fibroblasts, endothelial cells, pericytes, platelets, and macrophages (8). Tumor-associated macrophages (TAMs) represent an important component of the tumor micro- environment and can be divided into M1 and M2 types (9). A large number of studies have shown that M2 TAMs promote the occurrence and development of tumors by secreting vascular endothelial growth factor (VEGF), which then participates in angiogenesis. Further, matrix metalloproteinases (MMP2, MMP9), which promote tumor invasion and metastasis (10, 11), are significantly related to a poor tumor prognosis (pancreatic cancer) (12, 13). Notably, the activation of the PI3K/AKT/NF-κB-signaling pathway is conducive to M2 macrophage polarization and is involved in tumor progression and resistance to chemotherapy (14).

The intestinal microbiota constitutes the body’s normal intestinal microorganisms and is closely related to human health (15). Indeed, imbalance of the intestinal microbiota can lead to various diseases such as inflammatory bowel disease (16), obesity (17), diabetes (18), and even cancer (19). In recent years, there has been increasing interest in the relationship between the intestinal microbiota and tumors in the context of tumor treatment (20). Studies have indicated that the intestinal microbiota can mediate significant anti-tumor effects *via* modulating inflammationand restore immune functions (21). In this context, chemotherapy leads to dysregulation and damage to the intestinal microbiota, which is characterized by a reduction in beneficial lactic-acid-generating bacteria, such as *Enterococcus* and *Bifidobacterium*, and by an increase in the pathogens *Escherichia coli* and *Staphylococcus* (22, 23). For example, an imbalance in the intestinal microbiota reduces the effect of anti-PD1 treatment on patients with advanced cancer. The use of antibiotics can reverse this phenomenon by increasing the relative abundance of *Akkermansia muciniphila* (24), which can regulate the thickness of the intestinal mucosa and maintain the integrity of the intestinal barrier (25). Although no link has been reported between the gut microbiome and immunity in ovarian cancer patients, Studies have showed that when mice receive antibiotic treatment, the progression of xenograft ovarian tumors was delayed (26). Although there are reports that Traditional Chinese Medicine can treat diabetes (27), obesity (28), and colitis (29) by regulating the intestinal microbiota, the relationship between anti-tumor Traditional Chinese Medicine and the intestinal microbiota has not yet been reported. Here, we hypothesized that the effect of TPL on drug-resistant ovarian cancer may be related to its ability to improve the relative abundance of intestinal microbiota.

We previously showed that triptolide (TPL), one of the primary active ingredients of *Tripterygium wilfordii*, which is a Traditional Chinese Medicine that has been reported to be therapeutically efficacious in rheumatoid arthritis, inhibited the growth, invasion, and migratory capability of drug-resistant ovarian cancer cells (30) and reversed the resistance of ovarian cancer cells to cisplatin by inhibiting the phosphorylation of AKT (31). In this study, drug-resistant ovarian cancer cells (A2780/DDP) were used to investigate whether TPL inhibited the invasion and migration of drug-resistant ovarian cancer by inhibiting the polarization of M2 TAMs through the PI3K/AKT/NF-κB signaling pathway, and to explore the relationship between TPL and the intestinal microbiota.

## Materials and Methods

### Cell Lines and Culture

The human ovarian cancer cell line A2780/DDP (purchased from the Cell Bank of the Chinese Academy of Sciences, Shanghai, China) was cultured in RPMI 1640 medium (Gibco Life Technologies, Grand Island, NY, USA) supplemented with 10% fetal bovine serum (FBS) (Biological Industries, Israel), 0.2 µg/mL cisplatin (Hansoh Pharma, China), and 100 U/mL penicillin/streptomycin (Solarbio, China). The human acute leukemia mononuclear cell line THP-1 (Procell Life Science & Technology Co., Ltd.) was cultured in RPMI-1640 medium containing 10% FBS and 100 U/mL penicillin/streptomycin (Solarbio, China) in a 37°C incubator containing 5% CO_2_.

#### Morphological Observations

Cells in the logarithmic growth phase were seeded in a 6-well plate at a concentration of 5 × 105 cells/well. After 24 h of incubation, A2780/DDP cells were treated with varying concentrations of triptolide (TPL) (J&K Scientific Ltd, Cat. no.: T2899) (0, 6.25, 12.5, 25, 50, and 100 nM). After 24 h of treatment, we observed the growth and morphological changes of the cells under an inverted phase-contrast microscope (Olympus, Japan).

#### CCK8 Cytotoxicity Assay

We used the CCK8 assay to evaluate the inhibitory effect of TPL on A2780/DDP cells. We seeded 100 µL of A2780/DDP cells (10^5^ cells/mL) into 96-well plates and conducted a control culture or treatment with varying concentrations of TPL (6.25, 12.5, 25, 50, and 100 nM) for 24 h. We added 10 μL of CCK-8 solution to each well and incubated the wells for 1 h. The optical density of each well was measured at 490 nm using a Microplate Reader (Molecular Devices, CA, USA).

### *In Vitro* TAM Model

The *in vitro* TAM model was generated as follows. Briefly, THP1 cells (5 × 10^5^ cells/mL) were differentiated using 200 nM phorbol 12-myristate 13-acetate (PMA) (32) (Solarbio, Cat. no.: p6741) for different time-periods (0, 24, 48, 72, and 96 h); we observed the changes in cellular morphology under an inverted phase-contrast microscope. Differentiation of PMA-treated cells was enhanced after the initial 3-d impetus by removing the PMA-containing medium and then incubating the cells in fresh RPMI 1640 with 20 ng/mL recombinant human IL-4 (PeproTech, Cat. no.: 200-04] and 20 ng/mL recombinant human IL-13 (PeproTech, Cat. no.: 200-13) for different time-periods (0, 24, 48, and 72 h) The complete medium was then replaced and cultured for another 2 days, and the supernatant was collected for subsequent experiments.

#### Enzyme-Linked Immunosorbent Assay (ELISA)

We measured cytokine production in TAM supernatants using commercially available ELISA kits (IL-10, IL-12) according to the manufacturer’s instructions (Beijing 4A Biotech Co., Ltd).

#### Cell Proliferation Assay

TPL was diluted in cell culture supernatant to different concentrations (Co-c, 6.25, 12.5, 25, 50, and 100 nM, where Co-c refers to the cell supernatant collected above). We then measured the proliferative capability of A2780/DDP cells in the various groups treated with different concentrations of TPL for 24 h using the commercially available Cell-Light EdU Apollo567 *in vitro* kit according to the manufacturer’s instructions (RIBOBIO).

### Transwell Migration and Invasion Assay

We used a 24-well Boyden chamber (with 8-μm pore size; Corning Costar, USA) for the transwell migration assay. A2780/DDP cells (3 × 10^4^ or 6 × 10^4^) were loaded into the top of the 24-well migration chamber in 200 µL serum-free medium, and 700 µL of RPMI 1640 medium containing 20% FBS was added to the lower chamber to induce cell migration. Cells were incubated with a range of TPL concentrations (0, 6.25, 12.5, 25, 50, and 100 nM), or different concentrations of TPL (0, 6.25, 12.5, 25, 50 and 100 nM) diluted with cell culture supernatant or control in an incubator for 72 h. The cells that migrated into the lower surface of the filter were fixed with 4% paraformaldehyde for 20 min, washed 3 times with phosphate buffered saline (PBS), and then stained with 0.1% crystal violet solution for 30 min. Photomicrographs (100 ×) were taken with an Olympus IX51 (Olympus Optical, Melville, NY, USA) inverted microscope and three visual fields were counted. The sterile Borden chamber was also used for invasion measurements. BD Matrigel (BD Biosciences, USA) was placed in a −4°C refrigerator overnight before commencing the experiment. We placed the pipette tip and Boyden chamber in a refrigerator at −4°C 30 min before the start of the experiment, at which point the BD matrix liquified. We then diluted the BD matrix with serum-free RPMI 1640 medium at a 1:9 ratio. When the upper chamber had been pre-coated with Matrigel, we performed operations similar to the above migration analysis.

#### Extracellular Matrix-Adhesion Assay

A2780/DDP cells were seeded in 6-well plates, treated with different concentrations of TPL (0, Co-c, 6.25, 12.5, 25, 50, and 100 nM) and then transplanted to 12-well plates (1 × 10^5^ cells/well). The cells were cultured in an incubator at 37°C in an atmosphere of 5% CO_2_ for 3 or 6 h before adding the culture solution, and the unattached cells were aspirated at five replicates per group. The 6-well plate was washed twice with PBS, and all cells were collected after treatment with trypsin. We counted the number of cells to calculate the cellular adhesion rates using the following formula: cell adhesion rate = (cell adhesion number/total cell number) × 100%. All experiments were performed in triplicate.

### *In Vivo* Experiment

#### Establishment of the Xenograft Tumor Model

The A2780/DDP cells were cultured, and the densities were adjusted to 1 × 10^7^ cells/mL. We used inbred female BALB/c nude mice at 6 weeks of age and weighing 15–20 g that were raised under strict Specific Pathogen Free (SPF) conditions; all mice were provided with free access to water and chow. We then extracted 0.1 mL of cell suspension (containing 1 × 10^6^ cells) and inoculated the cells into the axilla of the mice using a 1-mL syringe. The control group (i.e., animals without cell injection) was created by administration of 0.1 mL of normal saline to the stomach once every 2 days for a total of 10 times. The tumor model (M) group was administered in the same manner as the control group. The cisplatin (DDP) treatment group was administered 4 mg/kg/d cisplatin intraperitoneally on days 1 and 8. The TPL treatment group received 0.15 mg/kg/d of triptolide diluted to a final volume of 0.1 mL with normal saline, and the drug was administered to the abdominal cavity once a day for 14 days. In addition, the TPL + DDP group was administered the combination of 0.15 mg/kg/d TPL to the abdominal cavity once a day for 14 days and 4 mg/kg/d DDP to the abdominal cavity on days 1 and 8.

#### Tumor Growth in Nude Mice

Following the establishment of our model, tumor volumes were measured by Vernier calipers every 2 days and tumors were photographed. On the 15th day, three mice in each group (except for the control group) were euthanized; their tumors were excised and frozen at −80°C. The tumors were then weighed, and the tumor inhibition rate was calculated. The remaining nude mice were used to generate survival curves to evaluate the survival of tumor-bearing mice.

#### Immunohistochemistry

After fixation, tumor tissues were embedded in paraffin, and tissues sections were cut at a 5-μm thickness and rehydrated in xylene (Sigma Cat. no.: 247642) and descending concentrations of 75% ethanol for 5 min, and then washed 4 times using PBS (5 min for each wash). Sections were incubated in 3% horse serum to block nonspecific binding. Sections were then incubated with anti-CD206 (Proteintech, Cat. no.: 18704-1-A P) and anti-CD31 (Affinity Biosciences, Cat. no.: AF6191 antibodies overnight at 4°C. Primary antibodies were detected by appropriate secondary antibodies; e.g., goat anti-rabbit secondary antibody (1:200; Servicebio, Cat. no.: GB23303) or goat anti-mouse secondary antibody (1:200; Servicebio, Cat. no.: GB23301).

#### Western Blotting

Cells were lysed on ice with Radio Immunoprecipitation Assay (RIPA) buffer containing 1 mM Phenylmethylsulfonyl fluoride (PMSF) to inhibit proteolysis. Equal amounts of proteins were separated by 10% SDS-PAGE and transferred onto polyvinylidene fluoride membranes (PVDF). The PVDF membranes were incubated with rabbit anti-MMP2 (1:1000; Proteintech, Cat. no.: 10373-2-AP), rabbit anti-Arg-1 (1:1000; CST, Cat. no.: 93668T), rabbit anti-MMP9 (1:1000; Proteintech, Cat. no.: 10375-2-AP), rabbit anti-VEGF (1:1000; Proteintech, Cat. no.: 19003-1-AP), rabbit anti-p65 (1:1000; Proteintech, Cat. no.: 10745-1-AP), rabbit anti-p-p65 (1:1000; CST, Cat. no.: 3033S), anti-AKT (1:1000; Proteintech, Cat. no.: 10176-2-AP), mouse anti-p-AKT (1:1000; Proteintech, Cat. no.: 66444-1-Ig), and mouse anti-β-actin (1:5000; Proteintech, Cat. no.: 60008-1-Ig) primary antibodies overnight at 4°C after blocking with 5% skim milk. Membranes were incubated with secondary antibodies for 2 h at room temperature, and proteins were visualized with an Ultra High Sensitivity ECL Kit.

#### High-Throughput 16S rDNA Gene-Amplicon Analysis

To elucidate the effects of TPL on the intestinal microbiota in nude mice, we collected fecal samples from the control (C) (n = 8), tumor model (M) (n = 7), DDP (n = 7), TPL (n = 7), and DDP + TPL (n = 7) groups for high-throughput sequencing. Paired-end reads from the original DNA fragments were handled using Cutadapt (version 1. 9. 1, http://cutadapt.readthedocs.io/en/stable/) and the UCHIME Algorithm (http://www.drive5.com/usearch/manual/uchime_algo.html). We subsequently performed sequence analysis using the UPARSE software package (version 7.0.100), and sequences with ≥ 97% sequence identity were assigned to the same operational taxonomic units (OTU). Qiime software (version 1.9.1) was used to analyze the diversity (within samples—indices of observed-OTUs, Chao1, Shannon, Simpson, ACE, and goods-coverage) and β diversity (among samples—PCA, principal coordinates analysis [PCoA], and NMDS). Cluster analysis was preceded by weighted UniFrac distancing using QIIME software (version 1.8.0), and partial least-squares discriminate analysis (PLS-DA) was performed using SIMCA-P software version 11.5 (Umetrics; Sartorius Stedim Biotech). The resulting high-throughput sequencing data have been uploaded to NCBI, with GenBank accession number PRJNA673986.

#### Statistical Analysis

All statistical analyses were performed using Prism 7 (GraphPad). The log-rank test and 1- or 2-way analysis of variance (ANOVA) followed by Tukey’s multiple-comparison test were used in all studies, as noted in the figure legends. Data are presented as mean ± standard deviation (SD). Statistical significance was defined as *P < 0.05, **P < 0.01, and ***P < 0.001.

#### Ethics Statement

This study was approved by the Ethics Committee of Nanchang Royo Biotech Co. Ltd (RYE2019062702), and all studies were conducted according to approved guidelines.

## Results

### TPL Inhibits the Survival, Migration, and Invasion of A2780/DDP Cells

**Figure 1:**
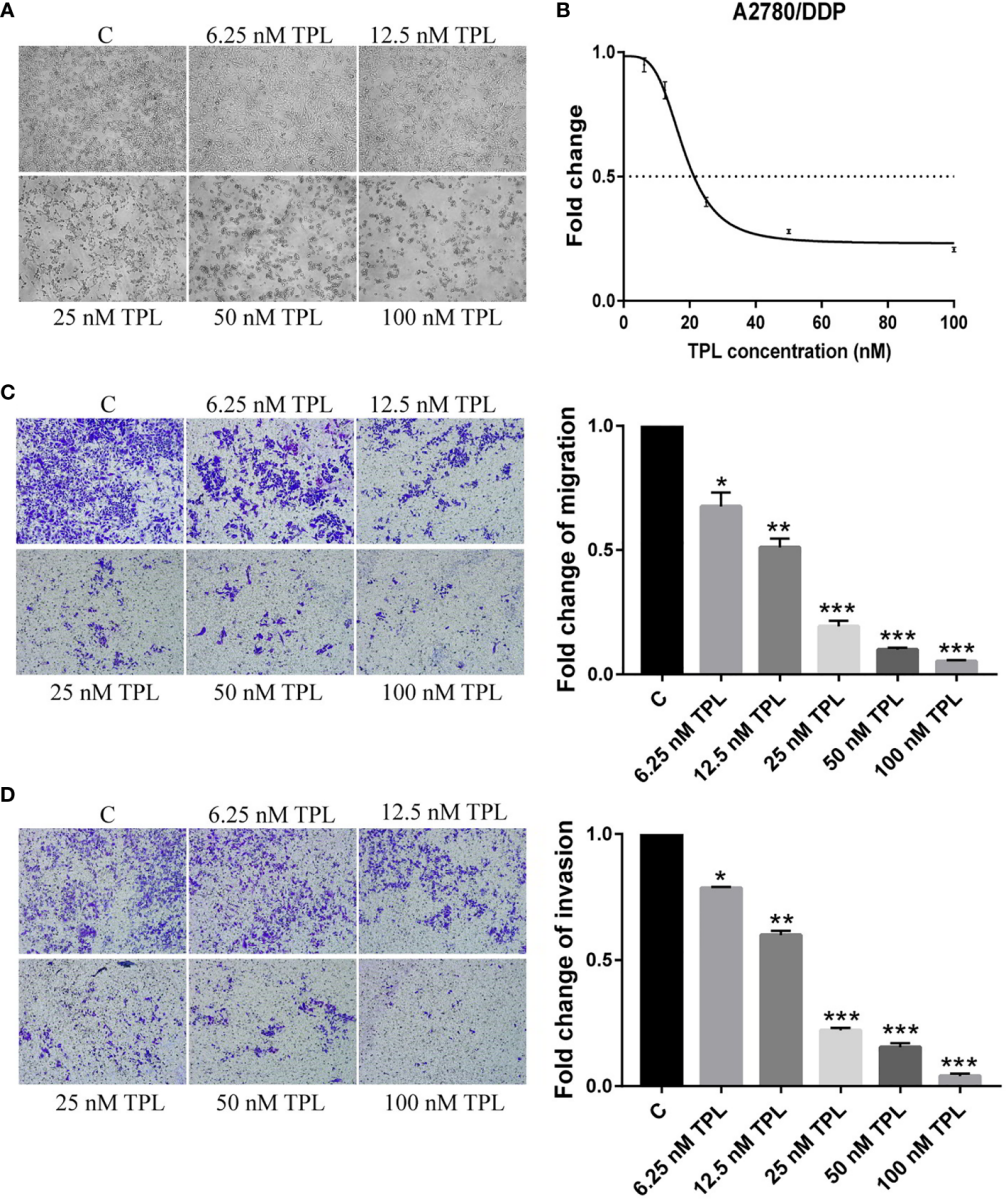
Triptolide (TPL) inhibits the survival, migration, and invasiveness of A2780/DDP cells. **(A)** A2780/DDP cells were treated with different concentrations of TPL for 24 h and photographed (× 100 magnification). **(B)** A2780/DDP cells were treated with a range of concentrations of TPL for 24 h to examine cellular survival. **(C,D)** Transwell migration and invasion assay of A2780/DDP cells after treatment with increasing concentrations of TPL (× 100 magnification). *P < 0.05, **P < 0.01, and ***P < 0.001.

The A2780/DDP cell line is a cisplatin-resistant human epithelial ovarian cancer cell line. In the current study, to examine the response of A2780/DDP cells to TPL, we used a series of different concentrations of TPL. After 24 h, the morphological changes of the cells were analyzed, as shown in **Figure 1A**. As the concentration of TPL increased, the cell density gradually decreased, and the cellular debris increased commensurately. We also performed a CCK8 cytotoxicity assay to evaluate the effect of TPL on the survival rate of A2780/DDP cells. We found that as the TPL concentration gradually increased, the optical density value was gradually reduced (**Figure 1B**), with the IC50 for TPL at 18.26 nM. Next, we used transwell invasion and migration experiments to investigate the effect of TPL on the migratory and invasive capabilities of A2780/DDP cells (**Figure 1C, D**). Compared to the control group, as the concentration of TPL increased, the migratory and invasive capabilities of the cells were gradually reduced in a dose-dependent manner. These data suggest that TPL can significantly inhibit the survival of A2780/DDP cells and downregulate the migration and invasion of tumor cells.

### Establishment of the TAM Model *In Vitro*

**Figure 2:**
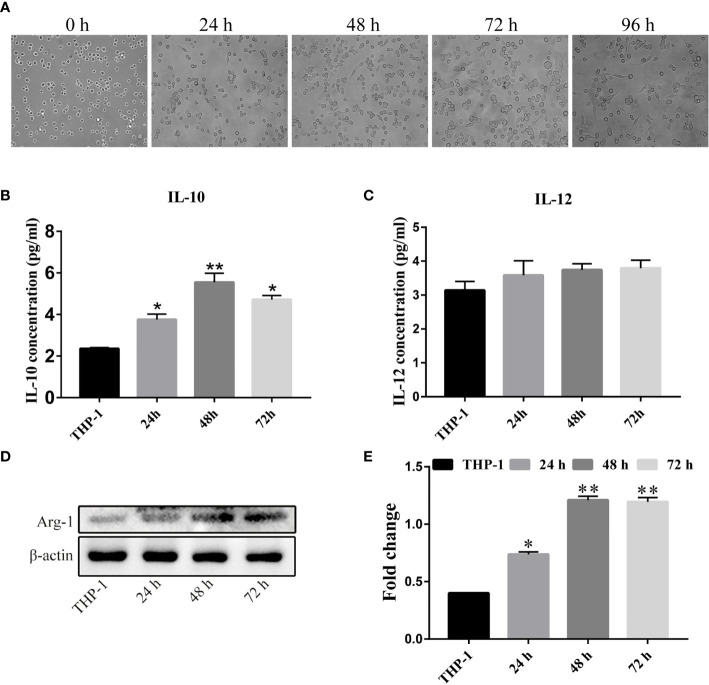
Establishment of a tumor-associated macrophage (TAM) model in *vitro*. **(A)** THP-1 cells were treated with 200 nM PMA for different time-periods (× 100 magnification). **(B, C)** THP-1 cells were transformed into M0 TAMs after PMA treatment, and were then treated with IL-4 and IL-13 for varying durations. Cell supernatants were extracted for ELISA to determine IL-10 and IL-12 levels. **(D, E)** The expression and quantitative analysis of Arg-1 protein in cells after the above treatment groups. The above results indicated that we successfully obtained M2-type macrophages after the above treatment. *P < 0.05, **P < 0.01.

We next established a TAM model *in vitro*. We seeded acute mononuclear leukemia cells (THP-1) in their logarithmic growth phase in a 6-well plate at 1 × 10^6/^mL and treated the cells with 200 ng/mL PMA for 24 h, 48 h, or 72 and 96 h. When we observed their cellular morphology (**Figure 2A**), we found that the cells were in a circular suspension state with almost no cellular attachment without PMA. However, after adding 200 ng/mL PMA as an inducer, in addition to extending the induction time, the proportion of adherent cells increased. After 72 h of induction, we uncovered the largest number of adherent cells, irregular shapes, and obvious filopodia. At this time, the irregular cells were M0 unpolarized macrophages. To induce polarization of M0 macrophages to form M2 macrophages, after 72 h of PMA treatment, we changed the complete medium containing 20 nM recombinant human IL-4, and 20 nM recombinant human IL-13, and continued to culture cells for 24 h, 48 h, and 72 h. Next, we detected the concentrations of IL-10 and IL-12 in the cell culture supernatant using the Human IL-10/IL-12p70 ELISA kit (**Figures 2B, C**). Compared to the THP-1 cell culture supernatant, IL-4 and IL-13 induced high levels of IL-10 in the cellular supernatant 48 h after treatment, but IL-12 did not. Significant differences are shown at the different time-points. Subsequently, we collected the above treatment group cell extraction and determined the total expression of Arginine Protease 1(Arg-1) protein (**Figures 2D, E**). Compared to THP-1 cells, the longer the cells were incubated with IL-4 and IL-13, the higher the expression of Arg-1 protein. We found an interesting phenomenon in that the level of Arg-1 did not increase when the incubation time was longer than 48 h to 72 h, which we speculated might be related to the depletion of cell culture medium nutrients. These results indicate that the M2 tumor-associated macrophage model was successfully established.

### TAM Cell Supernatant Slightly Improves the Proliferative, Migratory, and Invasive Potentials of A2780/DDP Cells, Which Can be Reversed by TPL

**Figure 3:**
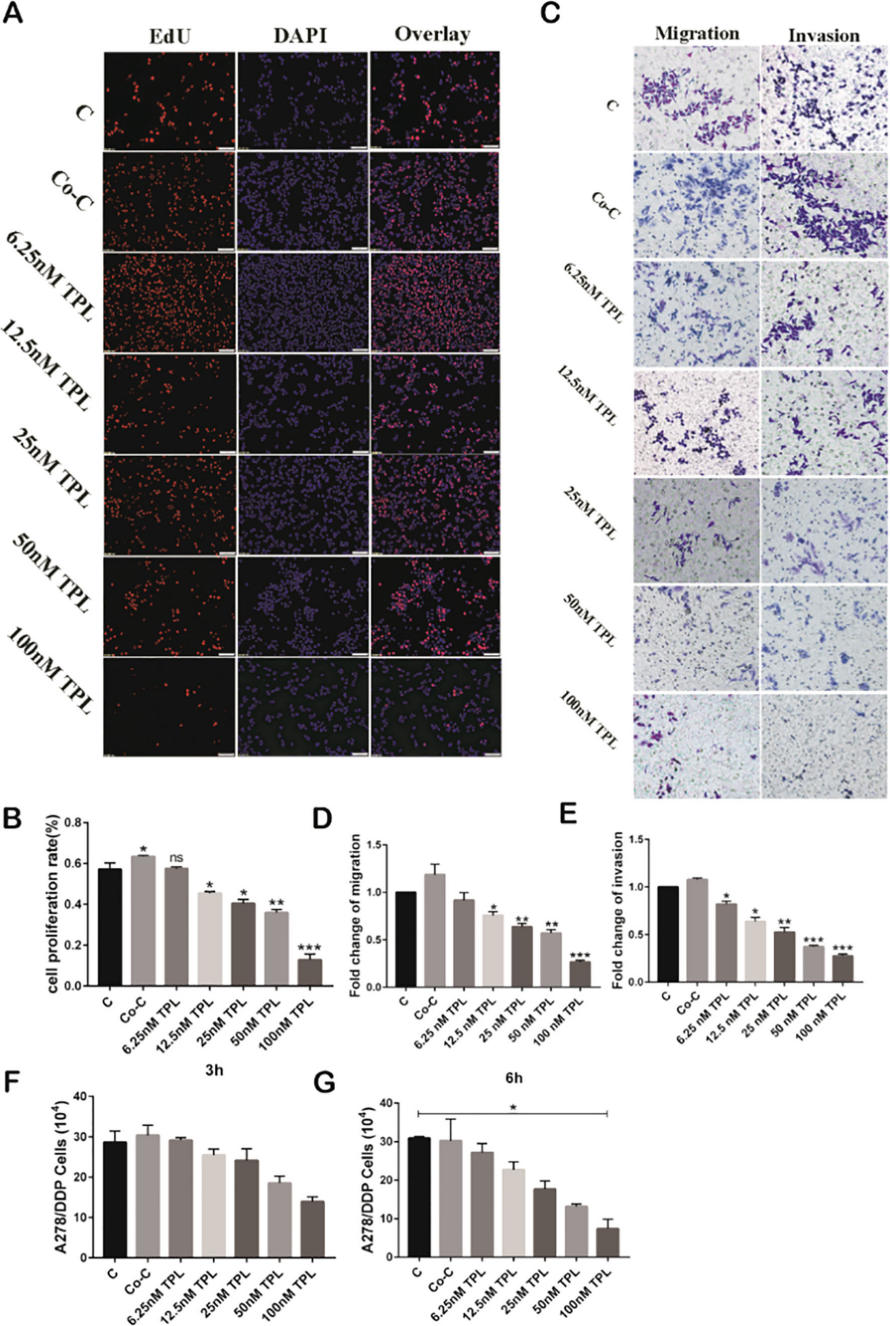
Tumor-associated macrophage (TAM) cell supernatant slightly improves the proliferation, migration, and invasiveness of A2780/DDP cells, but triptolide (TPL) reverses these effects. **(A)** TPL was diluted to varying concentrations with TAM cell supernatant, and then added to A2780/DDP cells; cell proliferative ability was measured 24 h later. **(B)** Quantitative analyses of the proliferative capacity of A2780/DDP cells are shown in **(A)**. **(C)** Representative transwell migration and invasion assay of A2780/DDP cells after treatment with TPL. **(D, E)** Quantification of migratory and invasive capacities of A2780/DDP in **(C)**. *P < 0.05, **P < 0.01, and ***P < 0.001. **(F, G)** Representative extracellular matrix-adhesion experiment using A2780/DDP cells treated with different concentrations of TPL for 3 or 6 h. *P < 0.05. ns, no significance.

To ascertain the actions of M2 TAMs on the biological behavior of A2780/DDP cells, we extracted supernatant from the M2 TAMs and used it to dilute TPL to various concentrations (Co-Culture [Co-C], 6.25, 12.5, 25, 50, and 100 nM). To first determine the effect of different concentrations of TPL on the proliferative ability of A2780/DDP cells, we implemented a cell proliferation assay, the results of which are shown in **Figures 3A, B**. We found that compared to the control group (cells treated with complete medium), the proliferative rate of A2780/DDP cells in the Co-C group was slightly increased; however, the cell proliferation rate gradually decreased commensurately with increasing TPL concentration. We next performed a transwell experiment to assess the effect of TAM supernatant on the migratory and invasive abilities of A2780/DDP cells (**Figures 3C–E**). The result of the transwell experiment was similar to that of the EdU experiment, with the number of A2780/DDP cells passing through the membrane found to be the largest in the Co-C group. However, as the concentration of TPL increased, the number of cells passing through the cell membrane gradually decreased in a dose-dependent manner. We also performed extracellular matrix-adhesion experiments, the results of which are shown in **Figure 3F**. We found that although there was no significant difference in the number of adherent cells in each treatment group 3 h after seeding, compared to the control group, the number of adherent cells in the co-culture group was slightly increased. We also found that as the concentration of TPL increased, the number of adherent cells showed a tendency to decrease (similar results at 6 h). Taken together, the above results indicate that TAMs can slightly improve cellular proliferation, migration, and invasion, and that TPL inhibits the proliferative, migratory, and invasive capabilities of A2780/DDP cells.

### TPL May Inhibit the Polarization of M2-Type TAMs Through the Inhibition of the PI3K/AKT/NF-κB Signaling Pathway

**Figure 4:**
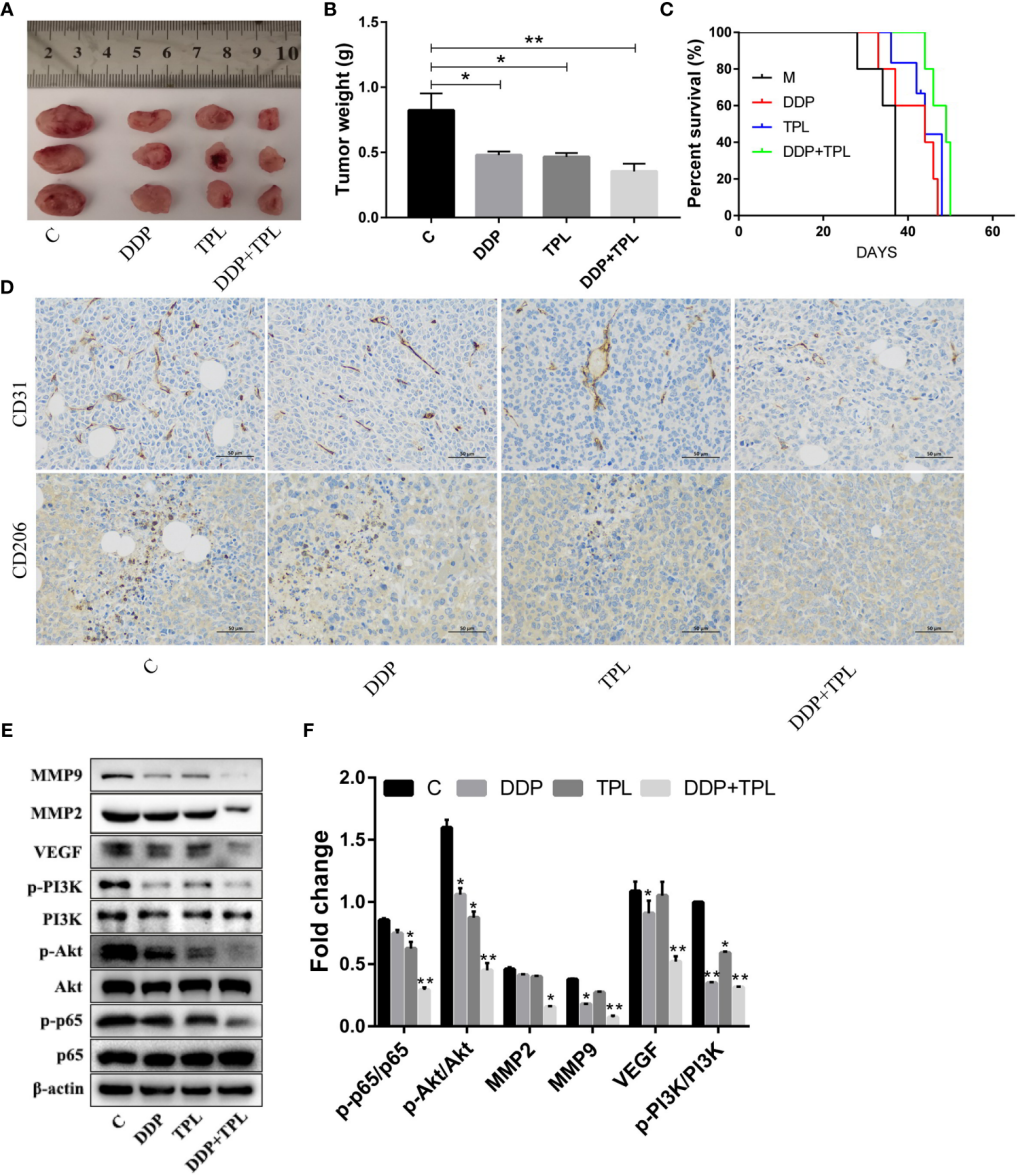
Triptolide (TPL) inhibition of the polarization induction of M2-type macrophages may be accomplished by inhibiting the PI3K/AKT/NF-κB pathway. **(A)** Images of tumors in the M, DDP, TPL, and DDP + TPL groups on day 15. **(B)** Weights (n = 3) of tumors in different treatment groups. **(C)** Kaplan–Meier survival curves of model mice bearing ovarian cancer after the intraperitoneal administration of TPL or DDP (n = 5). **(D)** Representative immunohistochemical expression of CD206 and CD31 in tumor tissues of different treatment groups. **(E, F)** Relative expression of MMP9, MMP2, VEGF, p-PI3K, PI3K, p-AKT, p-p65, AKT, and p65 in tumor tissues. *P < 0.05, **P < 0.01.

We shifted to using an *in vivo* experiment to further study the effect of TPL on drug-resistant ovarian cancer. DDP, TPL, or DDP + TPL was administered intraperitoneally to tumor cell-implanted mice to evaluate the effects on tumor growth and survival time. As shown in **Figures 4A, B**, compared to the control group, both DDP and TPL inhibited tumor growth (as shown by the reduction in tumor weight). The combination of DDP + TPL not only exerted the optimal inhibitory effect on tumor growth, but also significantly prolonged the survival time of tumor-bearing mice (**Figure 4C**). Our immunohistochemical results also showed that the expression of CD206 and CD31 was significantly inhibited by DDP + TPL (**Figure 4D**). Therefore, we posit that DDP + TPL effectively reduced the number of M2 macrophages in tumor tissues, and that TPL inhibited the expression of the vascular marker CD31, inhibiting angiogenesis in tumor tissues. Western blotting experiments showed that TPL, DDP, and DDP + TPL downregulated the levels of MMP-9, MMP-2, VEGF, p-PI3K, p-AKT, and p-P65 (**Figures 4E, F**). The above data showed that DDP + TPL can inhibit tumor invasion and migration, potentially by inhibiting the polarization of M2 macrophages through the PI3K/AKT/NF-κB- signaling pathway *in vivo*.

### Effects of DDP and TPL on the Intestinal Microbiota

**Figure 5:**
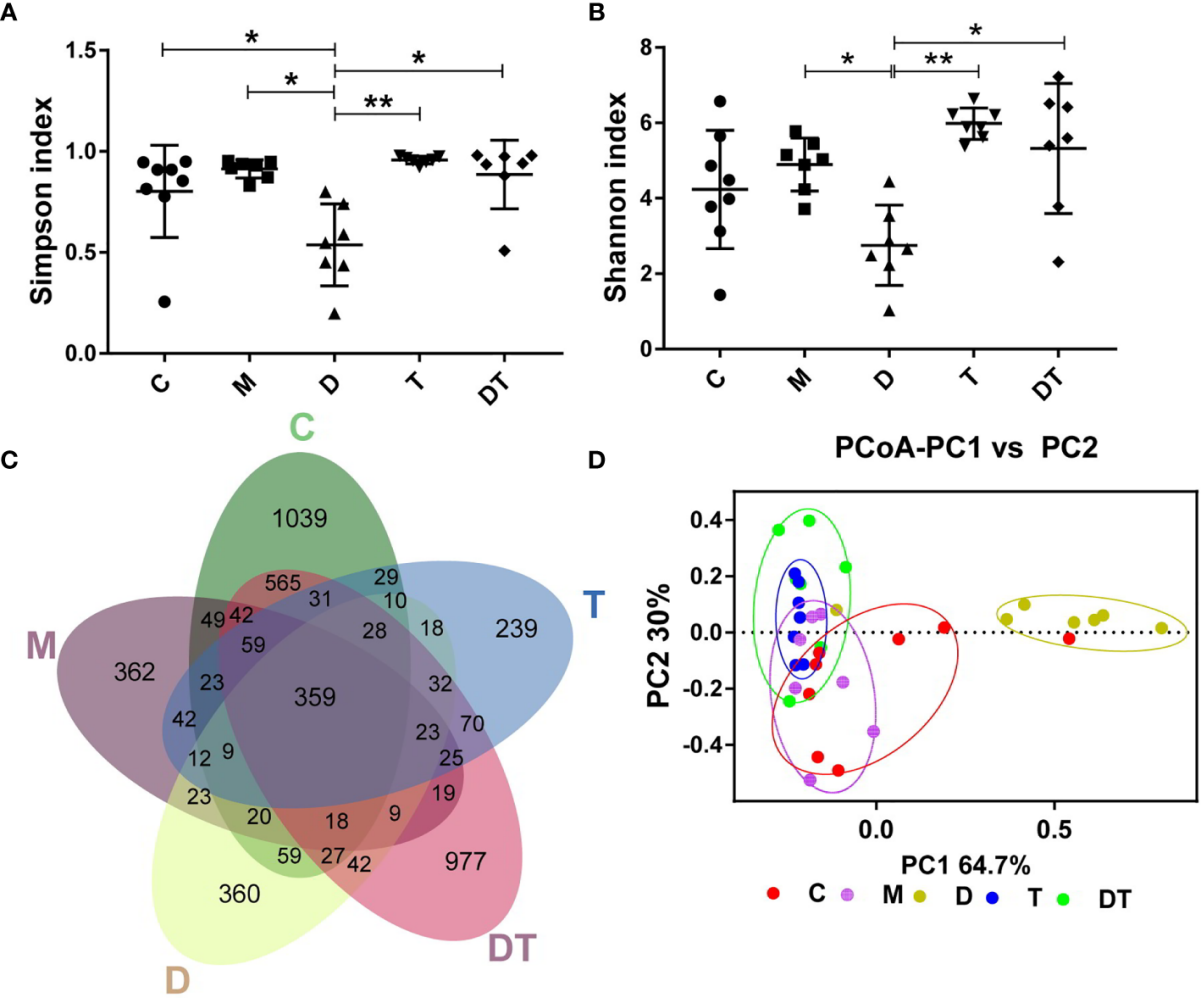
Effects of DDP and triptolide (TPL) on the intestinal microbiota of the tumor model. **(A)** The Simpson index, **(B)** the Shannon index, and **(C)** Venn map representing OTUs; **(D)** PCoA of the β diversity index. Data are presented as mean ± SD. One-way repeated-measures ANOVA with Tukey’s test used for multiple comparisons (**A–D**, respectively). These results indicated that DDP and TPL significantly altered the microbial compositions of nude mice.*P < 0. 05, **P < 0.01.

We next used high-throughput sequencing methods to investigate the effects of DDP and TPL on the intestinal microbiota of tumors in nude mice after ovarian cancer xenografts. A total of 14,826,581 filtered clean tags (411,849.5 tags/sample) and 15,807 OTUs were obtained from all the samples, with an average of 3161.4 OTUs per group (data not shown). Chao1 and observed species indices represent community diversity, while Shannon and Simpson indices represent total species; these indices were then used to evaluate the influence of DDP and TPL on the alpha diversity of the intestinal microbiota. We observed no significant changes in microbial abundance between different treatments (**Figure 5A**). Compared to the M groups, DDP significantly reduced microbial diversity, while TPL and DDP + TPL significantly improved the microbial diversity (**Figure 5B**). When analyzed using the Venn diagram method, 359 common OTUs were identified from all groups, and the unique OUT numbers in C, M, DDP, TPL, and DDP + TPL were 1039, 362, 360, 977, and 239, respectively (**Figure 5C**). PCoA analysis showed that dots were clustered in the TPL group and relatively dispersed in the DDP + TPL group. In addition, samples in the C and M groups manifested a close proximity, but remained far from the DDP group, indicating that the microbial diversity in the DDP group was obviously different from that in either the C or M group (**Figure 5D**).

**Figure 6:**
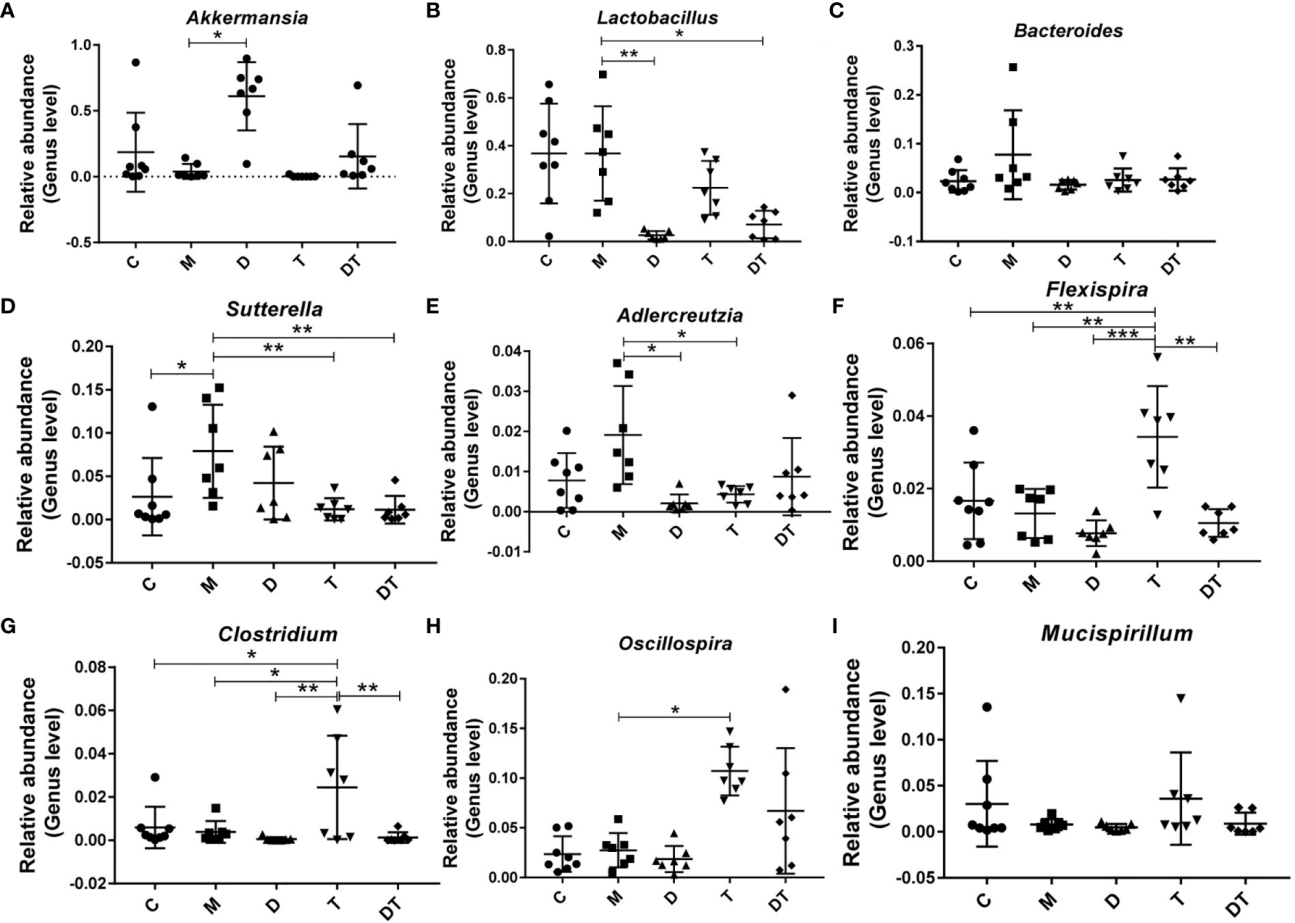
Influence of DDP and triptolide (TPL) on the abundances of different intestinal microbiota. The relative abundances of *Akkermansia* **(A)**, *Lactobacillus* **(B)**, *Bacteroides* **(C)**, *Sutterella* **(D)**, *Adlercreutzia* **(E)**, *Flexispira* **(F),** *Clostridium* **(G)**, *Oscillospira* **(H)**, and *Mucispirillum* **(I)** were analyzed. The above results suggested that DDP and TPL upregulate the abundance of beneficial bacteria and downregulate the abundance of harmful bacteria. Data are presented as the mean ± SD. One-way repeated-measures ANOVA was used with Tukey’s test for multiple comparisons (**A–I**, respectively). *P < 0.05, **P < 0.01, ***P < 0.01.

Next, when we selected the relatively abundant microbiota in the gut microbes of nude mice for analysis, our results indicated that the tumor model significantly increased the relative abundance of *Sutterella* (**Figure 6D**). Compared to the M group, treatment with DDP greatly increased the relative abundance of *Akkermansia* (**Figure 6A**) and reduced the relative abundances of *Lactobacillus* and *Adlercreutzia* (**Figures 6B, E**). Furthermore, treatment with TPL greatly increased the relative abundances of *Flexispira*, *Clostridium*, and *Oscillospira* (**Figures 6F–H**), and reduced the relative abundances of *Sutterella* and *Adlercreutzia* (**Figures 6D, E**). Intriguingly, although we noted few microbial changes in *Akkermansia*, *Bacteroides*, *Adlercreutzia*, *Flexispira*, *Clostridium Oscillospira*, or *Mucispirillum* between the M and DDP + TPL groups (**Figures 6A, C, E–I**), DDP + TPL reduced the relative abundances of *Lactobacillus* and *Akkermansia* (**Figures 6B, D**).

## Discussion

The high fatality rate associated with ovarian cancer and its resistance to existing chemotherapeutic drugs have made the development of anti-tumor drugs a top priority for clinical researchers. Considering the current difficulties in developing anti-tumor drugs, it is a reasonable option to extract anti-tumor drugs from Traditional Chinese Medicines. As one of the primary components of the Chinese herbal medicine *Tripterygium wilfordii* (a classic drug for treating rheumatoid arthritis), TPL has been found to offer broad-spectrum anti-tumor effects. Indeed, recent studies have shown that TPL reverses drug resistance in ovarian cancer. In the present study, we found that TPL inhibits the growth of drug-resistant ovarian cancer *in vivo* and *in vitro*, potentially by inhibiting the polarization of M2 TAMs through the PI3K/AKT/NF-κB-signaling pathway.

Previous studies have shown that TPL exerts powerful anti-tumor actions by inhibiting cellular proliferation (33), blocking the cell cycle (34), interfering with tumor angiogenesis (35), inducing autophagy, and promoting cellular apoptosis (36). We presented similar findings herein, where we determined that administering increasing concentrations of TPL to A2780/DDP cells commensurately augmented cellular apoptosis, elevated their cytotoxicity, and attenuated their ability for cellular migration and invasion (**Figure 1**). However, the underlying mechanism(s) of action remains unknown. It is evidence that M1 type macrophages secrete pro-inflammatory factors such as IL-12, which recognize tumor cells and play an important role in antigen presentation. Moreover, M2 macrophages secrete the cellular inflammatory factor IL-10 to regulate blood vessels, which, in turn, promotes the occurrence and development of tumors (37). Additionally, the characteristic factor Arg-1 of M2 macrophages was also significantly upregulated (**Figure 2**). We also found that when A2780/DDP cells were co-cultured with the supernatant from M2 TAMs, the proliferative ability of the cells was enhanced (**Figure 3**); compared to the control group, the invasive and migratory capabilities were also augmented. However, when TPL was added to the M2 TAM supernatant, the proliferative ability of A2780/DDP cells was inhibited in a dose-dependent manner; this coincides with the results of previous studies (38). M2 type macrophages participate in tumor angiogenesis through a variety of ways, including the release of a variety of matrix metalloproteinases, serine proteases, and cathepsins. The release of these factors contribution to destroy the endothelial cell basement membrane and decompose collagens and other components of the extracellular matrix, thereby aiding tumor and stromal cells in their migration (39). Western blotting (**Figure 4**) of nude mouse tumor tissues showed that TPL not only inhibited tumor growth, but also reduced the expression of MMP9, MMP2, VEGF, p-PI3K, p-AKT, and p-p65 protein. The immunohistochemical staining results additionally showed that TPL combined with DDP reduced the expression of CD206 and CD31, indicating that TPL inhibited tumor invasion and metastasis by inhibiting the expression of metalloproteinases and CD31. The inhibition of CD206 expression suggests that TPL inhibits the ability of M2 TAMs to undergo polarization, which may be accomplished by inhibiting the PI3K/AKT/NF-κB pathway. However, we have not examined the effect of TPL on the PI3K/AKT/NF-κB signaling pathway *in vitro*, nor have we specifically evaluated the regulation of TPL on the PI3K/AKT/NF-κB signaling pathway or determined how it affects the polarization of M2 TAMs, which are the limitation of our study.

Finally, high-throughput sequencing methods were used to detect microbial changes in the gut of nude mice. It appears that except for the closer microbiota of the M group and the C group, the microbiotas of the other treatment groups were obviously different. However, treatment with DDP significantly altered the microbial compositions (**Figure 5**). *Akkermansia* belongs to the genus *Ekmansia*, which is an intestinal symbiont that colonizes in the mucosal layer. *Akkermansia* not only participates in the immune regulation of the host, but also enhances the integrity of intestinal epithelial cells and the thickness of the mucous layer, thereby promoting intestinal tract health (40). A previous study showed an increase in the abundance of microbial in the intestines of patients with melanoma who were responsive to *Akkermansia*-combined immunotherapy, and the patient’s fecal bacteria were then applied to the mouse melanoma model with obvious tumor suppressed effects (41). *Clostridium butyricum* is one of the normal intestinal microbiota, and the butyric acid it produces is not only the main source of nutrition and energy for intestinal mucosa cells but can also repair damaged intestinal mucosa, which is beneficial for regulating the human intestinal microecological balance (42). Related studies have shown that *Clostridium butyricum* inhibits the development of intestinal tumors (43). Furthermore, *Sutterella*, which belongs to the phylum Proteobacteria, is a common symbiotic bacterium in the human intestinal tract. Previous studies have shown that the abundance of *Sutterella* microbiota is positively correlated with intestinal diseases (44). Therefore, the increased abundance of the intestinal beneficial bacteria *Akkermansia* and the reduced abundance of *Adlercreutzia* following treatment with DDP confirm the ability of DDP to change the composition of the intestinal microflora. Moreover, the anti-tumor effect of TPL may be related to the increase in *Clostridium* and the reduction in *Sutterella* and *Adlercreutzia* (**Figures 5** and **6**), but this hypothesis needs to be further tested.

In summary, our results indicate that TPL combined with DDP can decreased the polarization of M2-type TAMs, thereby suppressing the proliferation, migration, and invasiveness of A2780/DDP cells *in vitro*, significantly prolonging the survival time of nude mice; the mechanism may be realized by inhibiting the PI3K/AKT/NF-κB pathway. In addition, DDP combined with TPL was found to promote the abundance of the beneficial intestinal bacteria, *Akkermansia* and *Clostridium*, and reduce the relative abundance of the opportunistic pathogenic bacteria, *Sutterella* and *Adlercreutzia*. Collectively, our results indicate that TPL is a promising adjuvant chemotherapy drug that can be used in the clinical treatment of drug-resistant ovarian cancer.

## Data Availability Statement

The original contributions presented in the study are publicly available. This data can be found here: PRJNA673986.

## Ethics Statement

This study was approved by the Ethics Committee of Nanchang Royo Biotech Co. Ltd (RYE2019062702), and all studies were conducted according to approved guidelines.

## Author Contributions

FL and LY contributed equally to this work. TC, HH, and BT designed the current experiment. FL performed the experiments. FL, TC, LY, HH, BT, LY, YZ, FZ, YF, and YH analyzed all data and wrote this article. All authors contributed to the article and approved the submitted version.

## Funding

This work was supported by grants from the National Natural Science Foundation of China (No. 31860090, 81760729, 82060638), Jiangxi Provincial Department of Education (No. GJJ190024), Jiangxi Natural Science Foundation (No. 2017BAB215069, 20202BABL206102, 20202BAB216005), “double 10-thousand plan” of Jiangxi Province (innovation and technology professionals as the highend talent), and Department of health of Jiangxi Province (No. 202130359).

## Conflict of Interest

The authors declare that the research was conducted in the absence of any commercial or financial relationships that could be construed as a potential conflict of interest.

## Publisher’s Note

All claims expressed in this article are solely those of the authors and do not necessarily represent those of their affiliated organizations, or those of the publisher, the editors and the reviewers. Any product that may be evaluated in this article, or claim that may be made by its manufacturer, is not guaranteed or endorsed by the publisher.
