# Correction: TPL inhibits the invasion and migration of drug-resistant ovarian cancer by targeting the PI3K/AKT/NF-κB-signaling pathway to inhibit the polarization of M2 TAMs

**DOI:** 10.3389/fonc.2025.1641399

**Published:** 2025-09-08

**Authors:** Fuyin Le, Lilan Yang, Yiwen Han, Yanying Zhong, Fuliang Zhan, Ying Feng, Hui Hu, Tingtao Chen, Buzhen Tan

**Affiliations:** ^1^ Department of Obstetrics & Gynecology, The Second Affiliated Hospital of Nanchang University, Nanchang, China; ^2^ Department of Obstetrics & Gynecology, The First Affiliated Hospital of Nanchang University, Nanchang, China; ^3^ Institute of Translational Medicine, Nanchang University, Nanchang, China

**Keywords:** A2780/DDP cells, cisplatin (DDP) resistance, triptolide (TPL), PI3K/AKT/NF-κB- pathway, tumor-associated macrophages (TAMs)

There was a mistake in **Figure 3** as published. In **Figure 3C**, there are instances of duplicated areas between some images due to carelessness. Specifically, these occur between: Migration-C and Invasion-Co-C; Invasion-C and Migration-12.5nM TPL; Invasion-C and Invasion-6.25nM TPL; Invasion-Co-C and Invasion-12.5nM TPL; Invasion-6.25nM TPL and Migration-25nM TPL; Invasion-6.25nM TPL and Migration-12.5nM TPL. The corrected **Figure 3** appears below.

**Figure 3:**
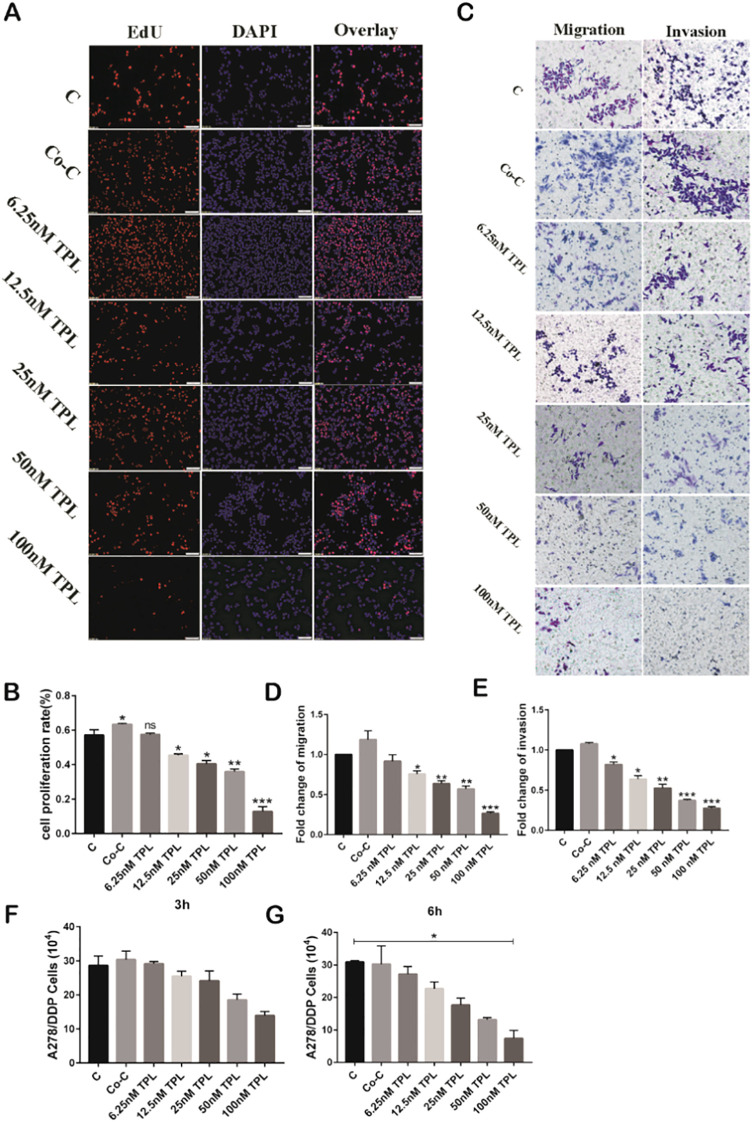
Tumor-associated macrophage (TAM) cell supernatant slightly improves the proliferation, migration, and invasiveness of A2780/DDP cells, but triptolide (TPL) reverses these effects. **(A)** TPL was diluted to varying concentrations with TAM cell supernatant, and then added to A2780/DDP cells; cell proliferative ability was measured 24 h later. **(B)** Quantitative analyses of the proliferative capacity of A2780/DDP cells are shown in **(A)**. **(C)** Representative transwell migration and invasion assay of A2780/DDP cells after treatment with TPL. **(D, E)** Quantification of migratory and invasive capacities of A2780/DDP in **(C)**. *P < 0.05, **P < 0.01, and ***P < 0.001. **(F, G)** Representative extracellular matrix-adhesion experiment using A2780/DDP cells treated with different concentrations of TPL for 3 or 6 h. *P < 0.05. ns, no significance.

The original version of this article has been updated.

